# Efficacy and safety of conventional disease-modifying antirheumatic drugs in VEXAS syndrome: real-world data from the international AIDA network

**DOI:** 10.3389/fphar.2025.1539756

**Published:** 2025-03-07

**Authors:** Antonio Vitale, Flavia Leone, Valeria Caggiano, Andrea Hinojosa-Azaola, Eduardo Martín-Nares, Guillermo Arturo Guaracha-Basañez, Jiram Torres-Ruiz, Perla Ayumi Kawakami-Campos, Pravin Hissaria, Alicia Callisto, Mark Beecher, Lorenzo Dagna, Alessandro Tomelleri, Corrado Campochiaro, Micol Frassi, Francesca Crisafulli, Franco Franceschini, José Hernández-Rodríguez, Verónica Gómez-Caverzaschi, Olga Araújo, Paolo Sfriso, Sara Bindoli, Chiara Baggio, Jessica Sbalchiero, Jurgen Sota, Abdurrahman Tufan, Ibrahim Vasi, Matteo Piga, Alberto Cauli, Maria Antonietta D’Agostino, Amato De Paulis, Ilaria Mormile, Henrique A. Mayrink Giardini, Rafael Alves Cordeiro, Francesco Gavioli, Giuseppe Lopalco, Florenzo Iannone, Carlomaurizio Montecucco, Sara Monti, Guillermo Ruiz-Irastorza, Adriana Soto-Peleteiro, Paola Triggianese, Carmelo Gurnari, Ombretta Viapiana, Riccardo Bixio, Rosetta Vitetta, Edoardo Conticini, Francesco La Torre, Gaafar Ragab, Ezgi Deniz Batu, Andrés González-García, Mercedes Peña-Rodríguez, Monica Bocchia, Ewa Wiesik-Szewczyk, Karina Jahnz-Rózyk, Alejandra de-la-Torre, Alberto Balistreri, Bruno Frediani, Claudia Fabiani, Luca Cantarini

**Affiliations:** ^1^ Department of Medical Sciences, Surgery and Neurosciences, Research Center of Systemic Autoinflammatory Diseases and Behçet’s Disease Clinic, University of Siena, Siena, Italy; ^2^ Azienda Ospedaliero-Universitaria Senese [European Reference Network (ERN) for Rare Immunodeficiency, Autoinflammatory and Autoimmune Diseases (RITA) Center] Siena, Siena, Italy; ^3^ UOC di Reumatologia, Fondazione Policlinico Universitario A Gemelli IRCCS, Università Cattolica del Sacro Cuore, Roma, Italy; ^4^ Department of Immunology and Rheumatology, Instituto Nacional de Ciencias Médicas y Nutrición Salvador Zubirán, Mexico City, Mexico; ^5^ Department of Ophthalmology, Instituto Nacional de Ciencias Médicas y Nutrición Salvador Zubirán, Mexico City, Mexico; ^6^ Department of Clinical Immunology and Allergy, Royal Adelaide Hospital, Adelaide, Australia; ^7^ Department of Immunopathology, SA Pathology, Adelaide, Australia; ^8^ Unit of Immunology, Rheumatology, Allergy and Rare Diseases, IRCCS Ospedale San Raffaele, [European Reference Network (ERN) for Rare Immunodeficiency, Autoinflammatory and Autoimmune Diseases (RITA) Center], Milan, Italy; ^9^ Università Vita-Salute San Raffaele, Milan, Italy; ^10^ Rheumatology and Clinical Immunology, Spedali Civili and Department of Clinical and Experimental Sciences, University of Brescia, [European Reference Network (ERN) for Rare Immunodeficiency, Autoinflammatory and Autoimmune Diseases (RITA) Center], Brescia, Italy; ^11^ Clinical Unit of Autoinflammatory Diseases, Department of Autoimmune Diseases, Institut d’Investigacions Biomèdiques August Pi I Sunyer (IDIBAPS), Hospital Clínic of Barcelona [European Reference Network (ERN) for Rare Immunodeficiency, Autoinflammatory and Autoimmune Diseases (RITA) Center], University of Barcelona, Barcelona, Spain; ^12^ Rheumatology Unit, Department of Medicine, University of Padua, [European Reference Network (ERN) for Rare Immunodeficiency, Autoinflammatory and Autoimmune Diseases (RITA) Center], Padua, Italy; ^13^ Department of Internal Medicine, Division of Rheumatology, Gazi University Hospital, Ankara, Türkiye; ^14^ Department of Medical Sciences and Public Health, University of Cagliari, Cagliari, Italy; ^15^ Rheumatology Unit, AOU Cagliari, Cagliari, Italy; ^16^ Department of Translational Medical Sciences, Section of Clinical Immunology, University of Naples Federico II, Naples, Italy; ^17^ Center for Basic and Clinical Immunology Research (CISI), WAO Center of Excellence, University of Naples Federico II, Naples, Italy; ^18^ Rheumatology Division, Faculdade de Medicina, Hospital das Clínicas, Universidade de São Paulo, São Paulo, Brazil; ^19^ Department of Precision and Regenerative Medicine and Ionian Area (DiMePRe-J) Policlinic Hospital, University of Bari, Bari, Italy; ^20^ Division of Rheumatology, Fondazione IRCCS Policlinico San Matteo, [European Reference Network (ERN) for Rare Immunodeficiency, Autoinflammatory and Autoimmune Diseases (RITA) Center], Department of Internal Medicine and Therapeutics, Università di Pavia, Pavia, Italy; ^21^ Faculty of Medicine and Nursery, University of the Basque Country, UPV/EHU, Biscay, Spain; ^22^ Autoimmune Diseases Unit, Biocruces Bizkaia Health Research Institute, Biscay, Spain; ^23^ Division of Internal Medicine-Hypertension, Department of Medical Sciences, Fondazione PTV Policlinico “Tor Vergata”, Rome, Italy; ^24^ Department of Biomedicine and Prevention, University of Rome Tor Vergata, Rome, Italy; ^25^ Department of Translational Hematology and Oncology Research, Taussig Cancer Institute, Cleveland Clinic, Cleveland, OH, United States; ^26^ Rheumatology Unit, Department of Medicine, University and Azienda Ospedaliera Universitaria Integrata of Verona, Verona, Italy; ^27^ Unit of Rheumatology, ASL VC Sant’ Andrea Hospital, Vercelli, Italy; ^28^ Department of Pediatrics, Giovanni XXIII Pediatric Hospital, University of Bari, Bari, Italy; ^29^ Rheumatology and Clinical Immunology Unit, Internal Medicine Department, Faculty of Medicine, Cairo University, Giza, Egypt; ^30^ Faculty of Medicine, Newgiza University, Giza, Egypt; ^31^ Department of Pediatric Rheumatology, Faculty of Medicine, Hacettepe University, Ankara, Türkiye; ^32^ Systemic Autoimmune Diseases Unit, Department of Internal Medicine, Hospital Universitario Ramón y Cajal, IRYCIS, Madrid, Spain; ^33^ Hematology Unit, Azienda Ospedaliera Universitaria Senese, University of Siena, Siena, Italy; ^34^ Department of Internal Medicine, Pneumonology, Allergology and Clinical Immunology, Central Clinical Hospital of the Ministry of National Defense, Military Institute of Medicine, National Research Institute, Warsaw, Poland; ^35^ Neuroscience Research Group (NEUROS), NeuroVitae Center, Escuela de Medicina y Ciencias de la Salud, Universidad del Rosario, Bogotá, Colombia; ^36^ Bioengineering and Biomedical Data Science Lab, Department of Medical Biotechnologies, University of Siena, Siena, Italy; ^37^ Ophthalmology Unit, Department of Medicine, Surgery and Neurosciences, University of Siena, Siena, Italy

**Keywords:** cDMARDs, clinical outcomes, autoinflammatory diseases, diagnosis, treatment

## Abstract

**Background:**

VEXAS (vacuoles, E1 enzyme, X-linked, autoinflammatory, somatic) syndrome is an adult-onset autoinflammatory condition resulting in severe, often treatment-refractory inflammation. Currently, there are no established treatment guidelines for VEXAS syndrome.

**Objectives:**

To assess the efficacy and safety of conventional disease-modifying antirheumatic drugs (cDMARDs) in a cohort of VEXAS patients.

**Methods:**

Data from VEXAS patients were obtained from the International AIDA Network VEXAS registry.

**Results:**

Data from 36 VEXAS patients were evaluated, with 28 (77.8%) treated with cDMARDs as monotherapy - and concomitant glucocorticoids (GC) - and 8 (22.2%) receiving a combination of different cDMARDs plus GC. Complete response (CR), partial response (PR), and failure to cDMARDs monotherapy were reported in 4/22 (18.2%), 11/22 (50%), and 7/22 (31.8%) courses, respectively. All patients were treated with GCs at the start of cDMARD monotherapy, and no GC discontinuation was observed later. No significant differences were observed in the GC dosage from the start of cDMARDs to the 3-month (*p* = 0.43), 6-month (*p* = 0.31), and 12-month (*p* = 0.21) visits. Conversely, the GC sparing resulted to be statistically significant when using methotrexate (*p* = 0.02). As for cDMARDs combinations, no cases achieved CR, while PR was observed in 5/9 (55.6%). Seventeen adverse events were reported, seven of which led to discontinuation.

**Conclusion:**

Many VEXAS patients report a partial benefit from cDMARDs, while a smaller yet not negligible number of patients exhibit a CR; cDMARDs remain a viable option for this disorder, especially when the initial GC dosage is low and the need for a steroid-sparing effect is not immediately urgent.

## Highlights

• Given the absence of standardized treatment guidelines for VEXAS syndrome, this study provides preliminary evidence on the therapy for this rare autoinflammatory condition
• This study evaluated the overall efficacy and safety profile of cDMARDs, administered either as monotherapy or in combination, in a substantial cohort of patients diagnosed with VEXAS syndrome
• This study highlights that cDMARDs achieved complete efficacy in only a minority of patients and demonstrated limited glucocorticoid-sparing effects; however, partial responses with effective control of specific clinical manifestations were common

## 1 Introduction

VEXAS (Vacuoles, E1 enzyme, X-linked, Autoinflammatory, Somatic) syndrome is an acquired monogenic autoinflammatory disease caused by somatic mutations in the *UBA1* gene. Discovered in 2020 by Beck et al. (Beck et al., 2020), VEXAS syndrome is characterized by severe systemic inflammation that can virtually affect all organs and tissues.

Regarding its etiopathogenesis, *UBA1* mutations lead to a decrease in ubiquitination and, consequently, to aberrant activation of the innate immune response (Beck et al., 2020). As the *UBA1* gene is located on the X chromosome, VEXAS syndrome primarily affects men. Due to the post-zygotic somatic onset of mutations, it generally manifests in adulthood, mainly after the age of 50. The most frequently reported *UBA1* gene mutations are missense mutations involving methionine at amino acid position 41, and they are found in hematopoietic progenitor cells.

The clinical presentation may be extremely heterogeneous, mimicking various other systemic rheumatologic entities coexisting with hematological disorders. Generally, VEXAS patients exhibit clinical features of autoinflammation, such as recurrent fever episodes, ear and nose chondritis, cutaneous vasculitis, neutrophilic dermatosis, pulmonary infiltrates, alveolitis, and venous thromboembolism, along with hematologic abnormalities including macrocytic anemia, thrombocytopenia, and signs of myelodysplastic syndrome (MDS) (Georgin-Lavialle et al., 2022; Gurnari et al., 2024a).

Prognostically, patients with VEXAS syndrome have an increased risk of morbidity and mortality, with a 5-year survival of approximately 80% and a median survival from onset of 10 years (Georgin-Lavialle et al., 2022; Ferrada et al., 2021; Gutierrez-Rodrigues et al., 2023).

To date, there are no standardized guidelines for managing this syndrome, and the available recommendations are based on a limited number of retrospective studies. The coexistence of inflammatory and hematologic symptoms in this condition often necessitates a multidisciplinary approach, and treatments must be tailored to each patient based on the predominant manifestation. In general, there are two main therapeutic strategies: inhibiting inflammatory pathways or cytokines through immunosuppressive drugs, cytokine inhibitors, and DNA methylation inhibitors, or eradicating progenitor stem cell clones carrying the *UBA1* mutation through hematopoietic cell transplantation (Nakajima and Kunimoto, 2024; Gurnari et al., 2024b). Certainly, glucocorticoids (GCs) have been the cornerstone of initial management for VEXAS syndrome, effectively alleviating inflammatory symptoms and cytopenia. However, medium to high doses are needed to achieve adequate control, necessitating alternative medications to reduce GC dosage and prevent long-term side effects.

Therefore, the purpose of this study is to evaluate the role of conventional disease-modifying antirheumatic drugs (cDMARDs) in patients with VEXAS syndrome based on real-world data gathered in the international AIDA Network VEXAS syndrome registry (Vitale et al., 2022).

## 2 Materials and methods

The main objective of this study was to assess the global efficacy of cDMARDs, used as monotherapy or in combination with other cDMARDs, in a relatively large cohort of patients diagnosed with VEXAS syndrome. An additional objective was to assess the safety profile of cDMARDs in these patients.

The endpoints for evaluating cDMARDs effectiveness were: i) the frequency of complete response, partial response, and treatment failure while on treatment; ii) the variations in daily prednisone (or equivalent) dosage from the start of cDMARDs’ treatment and the 3-, 6-, 12-month visit as well as the last assessment while on cDMARDs’ treatment.

All adverse events observed during the cDMARDs’ treatment regimen were reported to assess the safety profile.

Therefore, patients with VEXAS syndrome were consecutively enrolled from November 2021 to May 2024 in the international AIDA Network VEXAS syndrome registry. Data collection regarding treatment, clinical, and laboratory features was conducted both retrospectively (from disease onset to the time of enrollment into the AIDA registry) and prospectively (from the time of enrollment into the AIDA registry to the last follow-up assessment).

Laboratory assessments at onset and during follow-up included screening for hematologic abnormalities (anemia, leukopenia, thrombocytosis, and thrombocytopenia) and the inflammatory markers erythrocyte sedimentation rate (ESR) and C reactive protein (CRP). Reference ranges depended on the laboratories of the recruiting centers.

Inclusion criteria required the presence of a pathogenic or likely pathogenic mutation in the *UBA1* gene, along with the onset of a not otherwise explained systemic inflammatory condition. Signed informed consent for using clinical, laboratory, and genetic data was obtained in all patients enrolled.

The study was approved by the Ethics Committee of the Azienda Ospedaliero-Universitaria Senese, Siena, Italy, in June 2019 (Ref. N. 14951) as part of the AIDA Program. The study protocol conformed to the tenets of the Declaration of Helsinki.

Mutations in the *UBA1* gene, along with mutations associated with myelodysplastic syndrome or other oncohematological disorders, were detected through Next-Generation Sequencing or Sanger testing on peripheral blood or bone marrow samples.

Disease duration was defined as the period ranging from clinical onset to the start of each specific cDMARD. The follow-up period ranged from the initiation of cDMARDs to the last recorded assessment in the AIDA registry, with the index date corresponding to the start of cDMARD.

In the absence of standardized guidelines, physicians employed diverse treatment strategies based on their clinical expertise and the patient’s individual characteristics.

Regarding treatment outcomes, *complete response* (CR) was defined as the resolution of VEXAS-related clinical manifestations, accompanied by normalization or only a slight increase (no more than 10% above the upper limit of the cut-off) of inflammatory parameters (ESR and CRP) and no increase in the daily prednisone dosage. *Partial response* (PR) was defined as the persistence of clinical and laboratory manifestations with significantly reduced severity and/or frequency of acute exacerbations, as reported by patients and observed by physicians. *Failure* (F) was defined as the persistence of clinical manifestations and/or insufficient reduction in inflammatory markers to meet the previous definitions.

Both the terms “*cDMARDs monotherapy”* and “*combination of cDMARDs*” imply the concomitant use of glucocorticoids in all patients included in the analysis.

The term “adverse event” refers to any detrimental medical occurrence observed following exposure to any cDMARD treatment related to VEXAS syndrome, not necessarily caused by the treatment itself.

Regarding statistical analysis, descriptive statistics included percentages, mean, standard deviation (SD), median, interquartile range (IQR), and frequency counts as required. Qualitative data were analyzed using Fisher’s Exact test or Chi-squared test based on frequency counts and expected frequencies. Quantitative data were analyzed using Student's t-test or Mann-Whitney U test, depending on data distribution assessed through the Shapiro-Wilk test. Simple linear regression analysis was performed to search for any association between the cDMARDs discontinuation and the cDMARDs treatment duration (dependent variable) and the occurrence of withdrawal or the causes of withdrawal (independent variables). The significance level was set at 95% (*p*-value <0.05). The *p*-value was two-tailed in all cases. Statistical analysis was performed using RStudio software, version 4.3.0.

## 3 Results

At the start of the data analysis (27 May 2024), 71 patients were enrolled in the international AIDA registry dedicated to VEXAS syndrome. In detail, cDMARDs were used as monotherapy in 32 (45%) patients, as a combination of two cDMARDs in 7 (9.9%) cases, and as a combination of three cDMARDs in 1 (1.4%) case. Since the remaining patients used a combination of cDMARDs and biotechnological DMARDs, they were excluded from this study.

Regarding cDMARDs used as monotherapy, statistical analysis was performed on 41 treatment courses observed in 28 patients. Data from the remaining patients were only partial and erased (data missing at random). In total, 36 patients were included in the present study to assess the role of cDMARDs in VEXAS syndrome, either as monotherapy (28 patients) or in combination with other cDMARDs (8 patients). Table 1 provides demographic, clinical, and therapeutic information on the patients included in the study. The *UBA1* gene mutations identified in this cohort were: p.Met41Val in 10 (27.7%) cases, p.Met41Thr in 9 (25%) cases, p.Met41Leu in 4 (11.1%) cases; c.118-2A>G and c.118-1G>C mutations were identified in one patient each. Other 11 (30.6%) patients were reported to carry a *UBA1* mutation, which was not specifically specified in the registry.

**TABLE 1:** Demographic, clinical and laboratory features describing the patients treated with conventional disease modifying anti-rheumatic drugs (cDMARDs) in the present study, either as monotherapy or as cDMARDs combinations.

Clinical and laboratory features	
Sex, (females/males)	0/36
Age at disease onset, years (mean ± SD)	64.8 ± 12.7
Age at enrolment, years (mean ± SD)	71.8 ± 7.9
Fever during disease exacerbations, n (%)	26 (72.2)
Skin involvement, n (%)	27 (75)
Orbital/Ocular involvement, n (%)	19 (52.8)
Arthritis, n (%)	13 (36.1)
Gut involvement, n (%)	3 (8.3)
Neurological involvement, n (%)	6 (16.7)
Vessel involvement, n (%)	13 (36.1)
Kidney involvement, n (%)	2 (5.6)
Parenchymal lung involvement, n (%)	14 (38.9)
Pleuritis, n (%)	5 (13.9)
Pericarditis, n (%)	1 (2.8)
Orchitis, n (%)	3 (8.3)
Epididymitis, n (%)	3 (8.3)
Anaemia, n (%)	33 (91.7)
Leukopenia, n (%)	17 (47.2)
Thrombocytosis, n (%)	0
Thrombocytopenia, n (%)	16 (44.4)
Paraproteinemia, n (%) • *MGUS, n (%)* • *Other, n (%)*	3 (8.3) • 2 (5.6) • 1 (2.8)
Concomitant hematological disorders, n (%) • *Myelodysplastic syndromes* • *Myelodysplastic/myeloproliferative neoplasms*	34 (94.4) • 16 (44.4) • 1 (2.8)
Concomitant/Previous diagnosis of systemic inflammatory disease, n (%) • Relapsing polychondritis • Sweet syndrome • Seronegative Spondyloarthritis • Vasculitis • Systemic Lupus Erythematosus	20 (55.6) • 9 (25) • 2 (5.6) • 2 (5.6) • 4 (11.1) • 1 (2.8)

### 3.1 cDMARD monotherapy

Details about the cDMARDs employed in the 28 patients undergoing monotherapy are reported in Table 2. Data on treatment outcomes (CR, PR, and F according to the definitions provided in the Methods section) were available for 22 treatment courses and are also depicted in Table 2. A CR to cDMARDs monotherapy was reported in 4/22 (18.2%) courses. These were treated with azathioprine 75 mg/day in one case and with methotrexate in 3 cases, two of which initially administered 15 mg/week and later increased to 20 mg/week; the third case was treated with 15 mg/week, later increased to 25 mg/week.

**TABLE 2:** cDMARDs used as monotherapy included in the present study. This table depicts data on patients that underwent cDMARDs as monotherapy: the mean prednisone or equivalent dose at the start of cDMARD, the mean prednisone or equivalent dose at the last follow-up visit while on cDMARD and the overall response to the treatment are reported. Note that the treatment response, provided according to definitions reported in the Methods section, is available for 22 treatment courses; mean and standard deviations for glucocorticoid dosages at the start of cDMARDs and at the last assessment have been calculated based on data from 33 treatment courses. The decrease in the glucocorticoid dosage was statistically significant for methotrexate (*p* = 0.02), while the difference did not achieve significance for azathioprine (*p* = 0.17); this computation was not performed for other cDMARDs because of the small sample size. *Abbreviations: cDMARDs, conventional disease modifying anti-rheumatic drugs; CR, complete response; F, failure; GCs, glucocorticoids (prednisone or equivalent); NA, not available; PR, partial response.*

cDMARDs	Number of patients	GCs at the start (mg/day)	GC at the last follow-up visit	Response (N = 22)
Methotrexate	19	28.7 ± 18.9	15.2 ± 13.7	3 CR, 6 PR, 3 F
Azathioprine	9	32.5 ± 25.6	5.7 ± 1.15	1 CR, 1 PR, 1 F
Colchicine	4	16.7 ± 10.4	16.7 ± 10.4	1 PR, 1 F
Cyclosporine A	4	15 ± 10	7.5 ± 3.5	1 PR
Leflunomide	2	6.25 ± 1.77	12.3 ± 7.5	1 F
Hydroxycloroquine	1	25	NA	1 PR
Mesalazine	1	20	NA	1 PR
Mycophenolate Mofetil	1	NA	NA	1 F

A PR was reported in 11/22 (50%) courses: six treated with methotrexate (3 with 10 mg/week, one with 15 mg/week, one with 20 mg/week, one with no data regarding dose); one treated with hydroxychloroquine (400 mg/day); one treated with mesalazine (800 mg x3/day, later decreased to 400 × 3/day); one treated with cyclosporine (150 mg x2/day); one treated with azathioprine (100 mg/day); and one with colchicine (1 mg/day).

Failure was reported for 7/22 (31.8%) courses, as follows: three treated with methotrexate (10 mg/week later increased to 15 mg/week in one case, 10 mg/week in the second patient, 15 mg/week in the third patient), one treated with mycophenolate mofetil (dosage not provided), one case treated with leflunomide (dosage not provided), one treated with azathioprine (50 mg/day), and one treated with colchicine (1 mg/day).

The median duration of cDMARD treatment was 5 (IQR:11.25) months. At the time of data analysis, the current use of cDMARD was as follows: 16/28 (58.1%) patients discontinued after a median of 9 (9) months; 5/28 (17.9%) patients continued cDMARDs with biotechnological DMARDs; 6/28 (21.4%) patients continued cDMARDs alone; data for one patient was missing.

Data regarding the reasons leading to cDMARD withdrawal were available for 12 cases and were the following: no efficacy in 2 cases, poor efficacy in 2 cases, loss of efficacy in 2 cases, adverse events in 6 cases; there was no association between the duration of cDMARDs treatment and the withdrawal (linear regression model: β1 estimate = −10.36, p = 0.32) or the cause of withdrawal (β1 estimate from lack/loss of efficacy to adverse events = 14.00; p = 0.18).

Data on the use of GCs were available for 33 treatment courses and are depicted in Table 2; all patients were treated with GC at the start of cDMARD monotherapy, and no discontinuation was observed during the follow-up period. The median (IQR) prednisone equivalent dosage was 20 (17.5) mg/day at the start of cDMARDs, 15 (24.1) mg/day after 3 months, 21.25 (16.9) mg/day after 6 months, and 8.75 (4.38) mg/day at the 12-month assessment. No statistically significant differences were observed in the daily GC dosage from the start of cDMARDs to the 3-month (p = 0.43), 6-month (p = 0.31), and 12-month (p = 0.21) visits. Nine (27.3%) patients continued cDMARDs for more than 12 months; the decrease in GC dosage from the start of treatment to the last assessment was not statistically significant in these patients [from 30 (35) to 15 (35) mg/day, *p* = 0.18].

On the contrary, GCs sparing effect was statistically significant in the 19 cases treated with methotrexate, with a daily GC dosage of 28.7 ± 18.9 mg/day at the start of treatment and 15.2 ± 13.7 mg/day at the last assessment, after a median treatment duration of 6 (12.5) months (*p* = 0.02). The GCs sparing effect was not statistically significant for azathioprine (29.7 ± 21.4 versus 12.5 ± 10.9, p = 0.17); this aspect was not assessed for other cDMARDs because of the small sample sizes.

### 3.2 Combination of cDMARDs

As depicted in Table 3, a combination of cDMARDs was used in 8 cases, mainly as a combination of colchicine with another cDMARD: two patients with colchicine (1 mg/day) and methotrexate (15 mg/week), one patient with colchicine (0.5 mg/day) and hydroxychloroquine (200 mg/day), one patient with colchicine (0.5 mg/day) and leflunomide (20 mg/day). Other combinations included methotrexate (20 mg/week) plus hydroxychloroquine (300 mg/day), methotrexate (20 mg/week) plus azathioprine (50 mg/day), and methotrexate (10 mg/week) plus cyclosporine (200 mg/day), each used in one patient. The eighth patient received methotrexate (15 mg/week) plus hydroxychloroquine (200 mg/day) plus colchicine (1 mg/day).

**TABLE 3:** Treatment courses with conventional disease-modifying anti-rheumatic drugs (cDMARDs) combinations included in the present study.

Combination	Response	FU duration of combination (months)	GCs at start (mg/day)	GCs at last assessment (mg/day)	Notes
COL + MTX	PR	36	10	7.5	Colchicine withdrawn; MTX was continued as monotherapy as no additional efficacy had been obtained after combination
COL + MTX	F	NA	NA	NA	Both withdrawn due to lack of efficacy, changed to filgotinib
COL + HCQ	F	2	NA	NA	Both withdrawn due to lack of efficacy, changed to anakinra
COL + LEF	PR	7	50	0	Both have been maintained up to the last assessment
MTX + HCQ	F	22	5	5	Methotrexate has been withdrawn due to lack of efficacy
MTX + AZA	PR	20	25	25	Both withdrawn due to worsening anemia
MTX + CSA	PR	4	5	12.5	Both withdrawn due to loss of efficacy
MTX + HCQ + COL	PR	28	25	5	MTX withdrawn due to loss of efficacy

Each row refers to a specific cDMARDs combination used in eight different patients. The response defined according to definitions provided in the Methods section, the treatment duration and the glucocorticoids dosage (prednisone or equivalent) used at the start of the combination treatment and at the last assessment while on that combination are also provided. Abbreviations: AZA, azathioprine; COL, colchicine; CR, complete response; CSA, cyclosporine A; F, failure; FU, follow-up; GCs, glucocorticoids (prednisone or equivalent); HCQ, hydroxychloroquine; LEF, leflunomide; MTX, methotrexate; NA, not available; PR, partial response.

No cases achieved a complete response, while partial response was observed in 5/8 (62.5%) cases, as detailed in Table 3 along with other treatment specifics.

### 3.3 Safety profile

Among the 36 patients who received at least one course of cDMARDs (either as monotherapy or in combination), 17 AEs were observed in 12 (33.3%) patients throughout the follow-up period. In 7 out of 17 cases, AEs led to treatment discontinuation. Adverse events consisted of cytopenia (n = 9), gastrointestinal intolerance (n = 6), skin reaction (n = 1), and cutaneous abscess (n = 1). Adverse events are specifically illustrated in Table 4.

**TABLE 4:** Adverse events recorded during conventional disease modifying anti-rheumatic drugs (cDMARDs) treatment follow-up, considering cDMARDs monotherapy and combination therapy.

Adverse events	cDMARDs
Cytopenia ‐ Anaemia/worsening of anaemia ‐ Neutropenia ‐ Leukopenia ‐ Pancytopenia	‐ MTX (n = 3; 2 leading to discontinuation), Aza (n = 1, leading to discontinuation) ‐ MTX (n = 1), Aza (n = 2; 1 leading to discontinuation) ‐ MTX (n = 1) ‐ Leflunomide (n = 1, leading to discontinuation)
Gastrointestinal intolerance	- MTX (n = 4, 2 of which lead to discontinuation) - Colchicine (n = 2)
Skin reaction (dermatitis and eczema)	MTX (n = 1)
Cutaneous abscess	MTX (n = 1)

In the left column, the specific adverse events reported in the registry are listed; in the right column, the number of patients experiencing these adverse events is stratified according to the cDMARDs used, including the cases that led to discontinuation. Abbreviations: MTX, Methotrexate; Aza, Azathioprine.

AEs occurred in 4/16 patients with myelodisplastic syndrome and 8/20 patients without myelodisplastic syndrome (*p* = 0.48); peripheral blood count alterations occurred during cDMARDs treatment in 2/16 patients with myelodisplastic syndrome and 5/20 patients without myelodisplastic syndrome (*p* = 0.43).

## 4 Discussion

Currently, there are no established treatment guidelines for this newly recognized clinical entity, as no randomized controlled trials have been conducted. Consequently, managing VEXAS syndrome relies on clinical experience from other autoinflammatory conditions and insights from recently published case series and case reports.

Since immunologic studies of patient samples and zebrafish models in VEXAS syndrome demonstrated upregulation of multiple cytokine signatures including TNF, IL-6 and IFN-γ (Beck et al., 2020), a broad therapeutic approach could be beneficial rather than the specific blockade of single cytokines.

According to the limited data based on case series, cDMARDs are mostly effective temporarily and do not exhibit significant steroid-sparing effect in VEXAS syndrome (Bourbon et al., 2021; Koster et al., 2021; van der Made et al., 2022; Patel and Young, 2022; Boyadzhieva et al., 2023). In our study a complete response could be achieved in less than one-fifth of the patients treated with cDMARDs as monotherapy and none requiring a combination of cDMARDs, likely due to more severe disease activity in this group. About half of the patients reported some improvement in either the severity of symptoms or the frequency of inflammatory exacerbations, both in the monotherapy group and in those receiving cDMARDs’ combination therapy. Conversely, at least 30% of VEXAS patients did not experience any therapeutic benefit from cDMARDs. Overall, these results suggest that cDMARDs may have only a minor effect in patients with VEXAS syndrome. Furthermore, our findings confirm the poor GC-sparing effect. In this regard, discontinuing systemic steroids was nearly impossible while using cDMARDs, and the daily dosage of GCs did not decrease throughout the follow-up period in a significant fashion. An exception was observed in the subgroup of patients treated with methotrexate, who showed a statistically significant halving of the daily GCs dosages. This may suggest a more substantial role of methotrexate, but not enough sample sizes were available to assess this aspect for other cDMARDs.

Most patients treated with cDMARDs had to discontinue the treatment partially due to safety issues and partially due to lack or loss of efficacy or poor clinical benefit. However, there was no association between the duration of cDMARDs treatment and the likelihood of discontinuation. This finding indicates that both inefficacy/loss of efficacy and safety concerns equally impacted treatment persistence, *i.e.*, treatment discontinuation was not preferentially related to efficacy or safety issues. Similarly, no statistically significant association was observed between treatment duration and cDMARDs withdrawal, indicating that treatment discontinuation equally occurred during the entire follow-up period. Therefore, the occurrence of discontinuation was not polarised either in the initial months or in later stages, suggesting that efficacy or safety issues could arise with equal probability both in the first weeks of treatment and in subsequent phases.

Overall, according to our results and in line with the literature, the therapeutic role of cDMARDs appears quite poor in patients with this condition. However, since no treatment has proven definitively effective in VEXAS syndrome, cDMARDs may be considered a potential option and could be used as a therapeutic trial when other treatments are not feasible or effective.

The study’s limitations include the relatively small number of enrolled patients and the nature of data collection, as a registry can lead to various interpretations of results across different centers worldwide. Although these results need confirmation from future studies with larger cohorts to better assess the role of cDMARDs at varying dosages and to distinguish among different cDMARDs, the present study provides valuable evidence from real-life data collected from a consistent number of patients. Moreover, it would be helpful to assess the GCs sparing effect of cDMARDs other than methotrexate, but this will require larger sample sizes. Actually, response criteria were intentionally chosen with a relatively low stringent approach, including the absence of a mandatory reduction in glucocorticoids dosage. This decision was driven by the limited available knowledge on the real effectiveness of cDMARDs in patients with VEXAS syndrome and the recognized challenge of achieving a complete response when requiring a combination of symptoms resolution, normalization of laboratory parameters, and steroid dose reduction. Notably, attaining clinical and laboratory control without increasing a previously inadequate steroid dosage represents *per se* a meaningful achievement.

In conclusion, many VEXAS patients report a partial benefit from cDMARDs, while a smaller yet not negligible number of patients may exhibit a complete response. Therefore, given the limited treatment strategies currently available, cDMARDs may represent a therapeutic option to consider for VEXAS syndrome, particularly when the initial glucocorticoid dosage is low and the need for a steroid-sparing effect is not immediately urgent.

## Group member of from the International AIDA (AutoInflammatory Diseases Alliance) Network and the Autoinflammatory Diseases Working Group of the Italian Society of Rheumatology (SIR)

The authors of this publication [Antonio Vitale, Valeria Caggiano, Jessica Sbalchiero, Edoardo Conticini, Lorenzo Dagna, Alessandro Tomelleri, Corrado Campochiaro, Micol Frassi, Francesca Crisafulli, Franco Franceschini, Jurgen Sota, Francesco Gavioli, José Hernández-Rodríguez, Verónica Gómez-Caverzaschi, Olga Araújo, Paolo Sfriso, Sara Bindoli, Chiara Baggio, Carlomaurizio Montecucco, Sara Monti, Monica Bocchia, Alberto Balistreri, Bruno Frediani, Claudia Fabiani and Luca Cantarini] belong to institutes that are members of the ERN RITA [Azienda Ospedaliero-Universitaria Senese of Siena; Università Vita-Salute San Raffaele; University of Brescia; University of Padua; University of Pavia; Hospital Clínic of Barcelona].

## Data Availability

The raw data supporting the conclusions of this article will be made available by the authors, without undue reservation. Requests to access these datasets should be directed to the corresponding author.
